# Supplementary figures and images for: Machine learning prediction of non-attendance to postpartum glucose screening and subsequent risk of type 2 diabetes following gestational diabetes
Source: PLoS One. 2022 Mar 7;17(3):e0264648. doi: 10.1371/journal.pone.0264648 (PMC8901061; doi:10.1371/journal.pone.0264648)

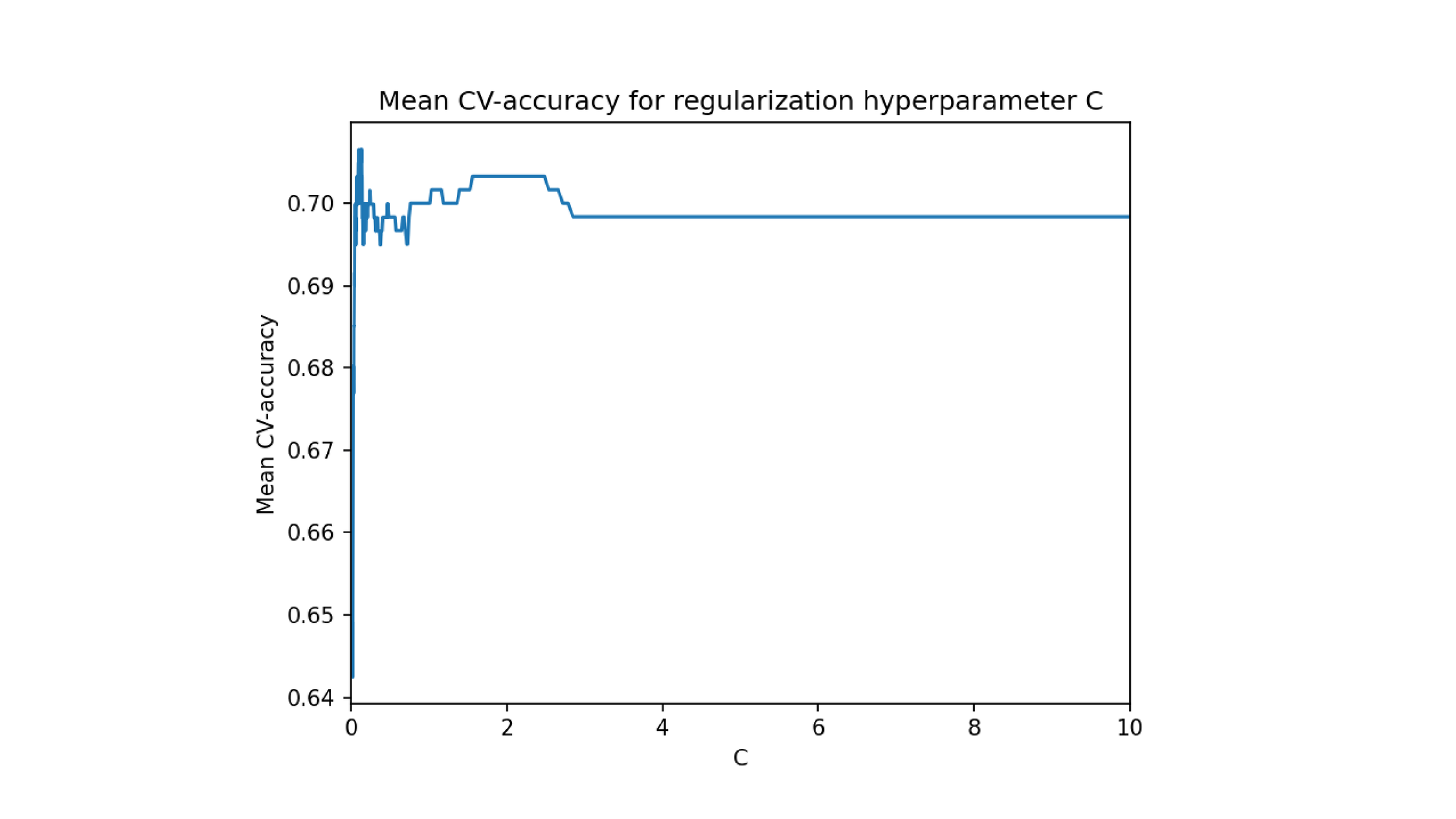

Supplement: S1 Fig — shows the variation in the mean stratified 10-fold cross validation accuracy as a function of the lasso regularization hyperparameter C for the final model. Maximum CV-accuracy of 0.7065 is obtained for C = 0.1325. (TIF) [file pone.0264648.s001.tif]

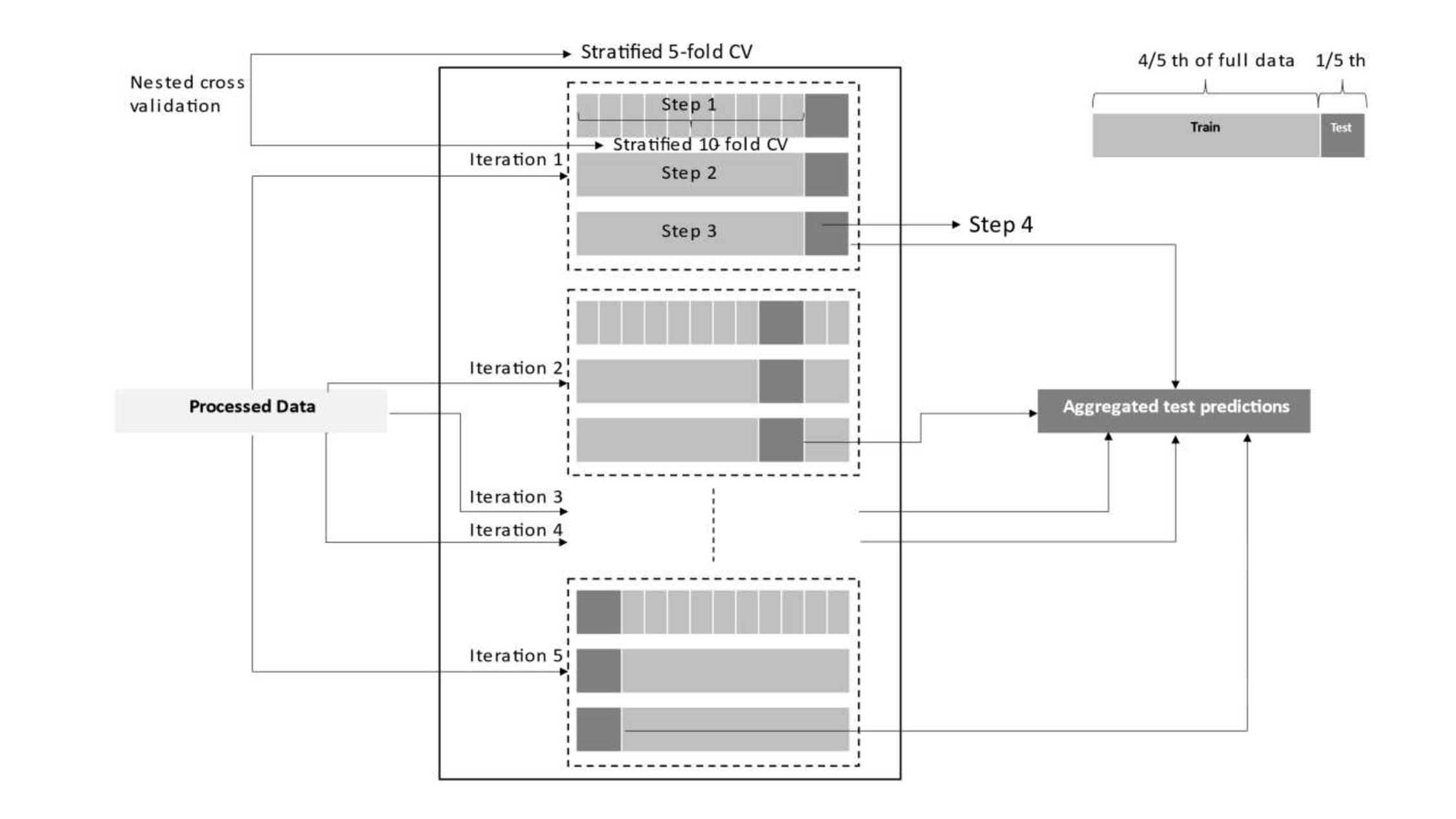

Supplement: S2 Fig — The processed data is divided into 5 folds. The light grey region shows the training data, and the dark grey shows the testing data. Within each iteration i of the outer 5-fold CV, the training folds further undergo internal 10-fold CV in Step 1 for lasso hyperparameter optimization. This is known as nested cross validation. In step 2, feature selection is performed on the training folds in iteration i using lasso with optimized regularization hyperparameter. Logistic regression model with selected features is fit on the training folds in iteration i in Step 3. The fit model is used for prediction on the exclusively held out test data in iteration i. The test predictions from all 5 iterations are aggregated to plot and calculate the area under the ROC curve for evaluating the performance of our method. (TIF) [file pone.0264648.s002.tif]

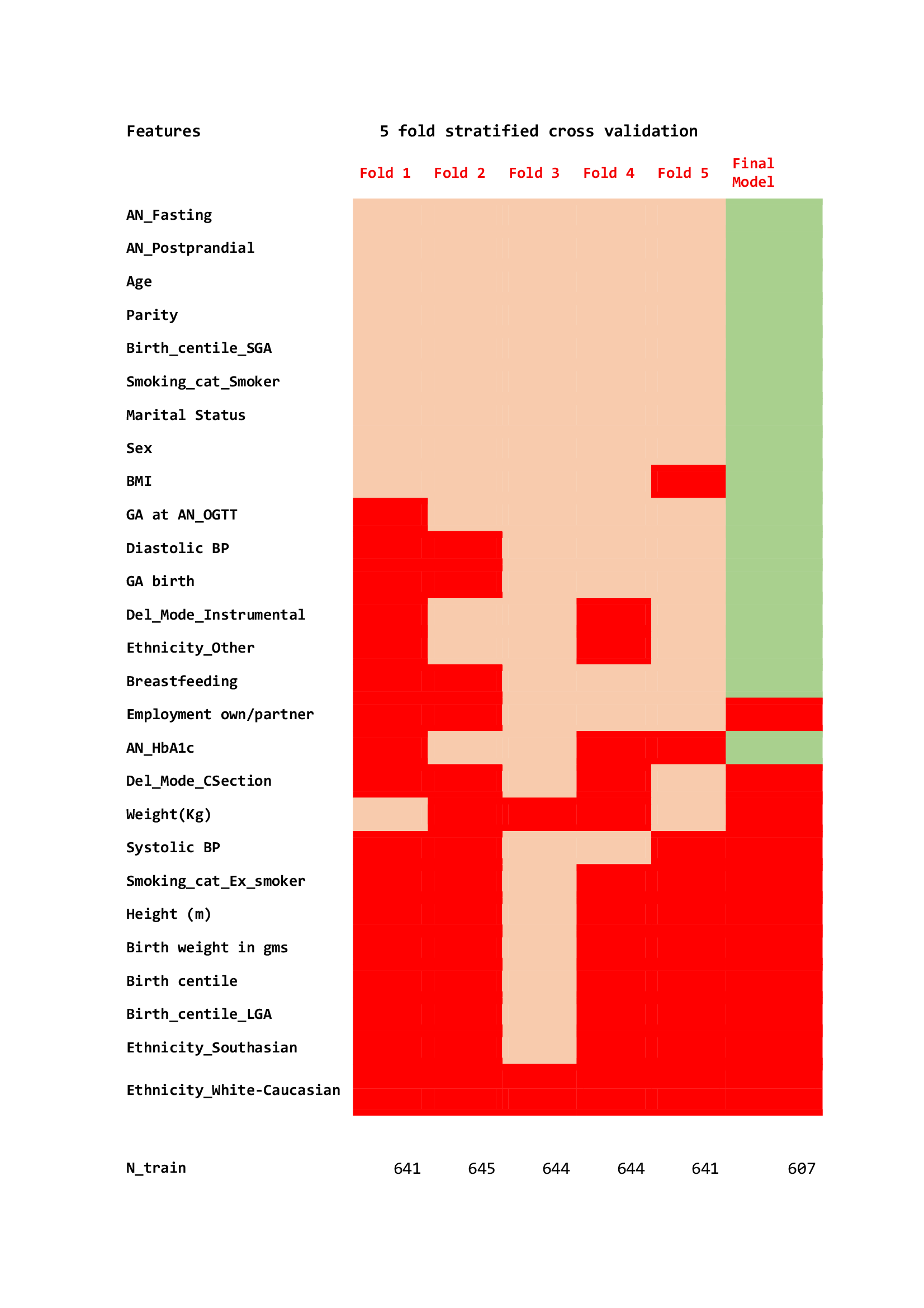

Supplement: S3 Fig — gives a visual understanding of the features selected in each iteration of CV1 and as well as in the final model. Pink cells show the selected features in CV1, green cells show the selected features in the final model built on the full data and red cells show the feature coefficients shrank to zero in both CV1 and the final model. The sets of features selected in all folds of CV1 (except fold 3, where all features except two are selected) and those selected in the final model are similar. The number of samples in the training and testing folds, value of the optimized hyperparameter C, number of features selected and area under the ROC curve for the test fold for each iteration i of CV1 as well as for the final model are provided below each respective column. (TIF) [file pone.0264648.s003.tif]

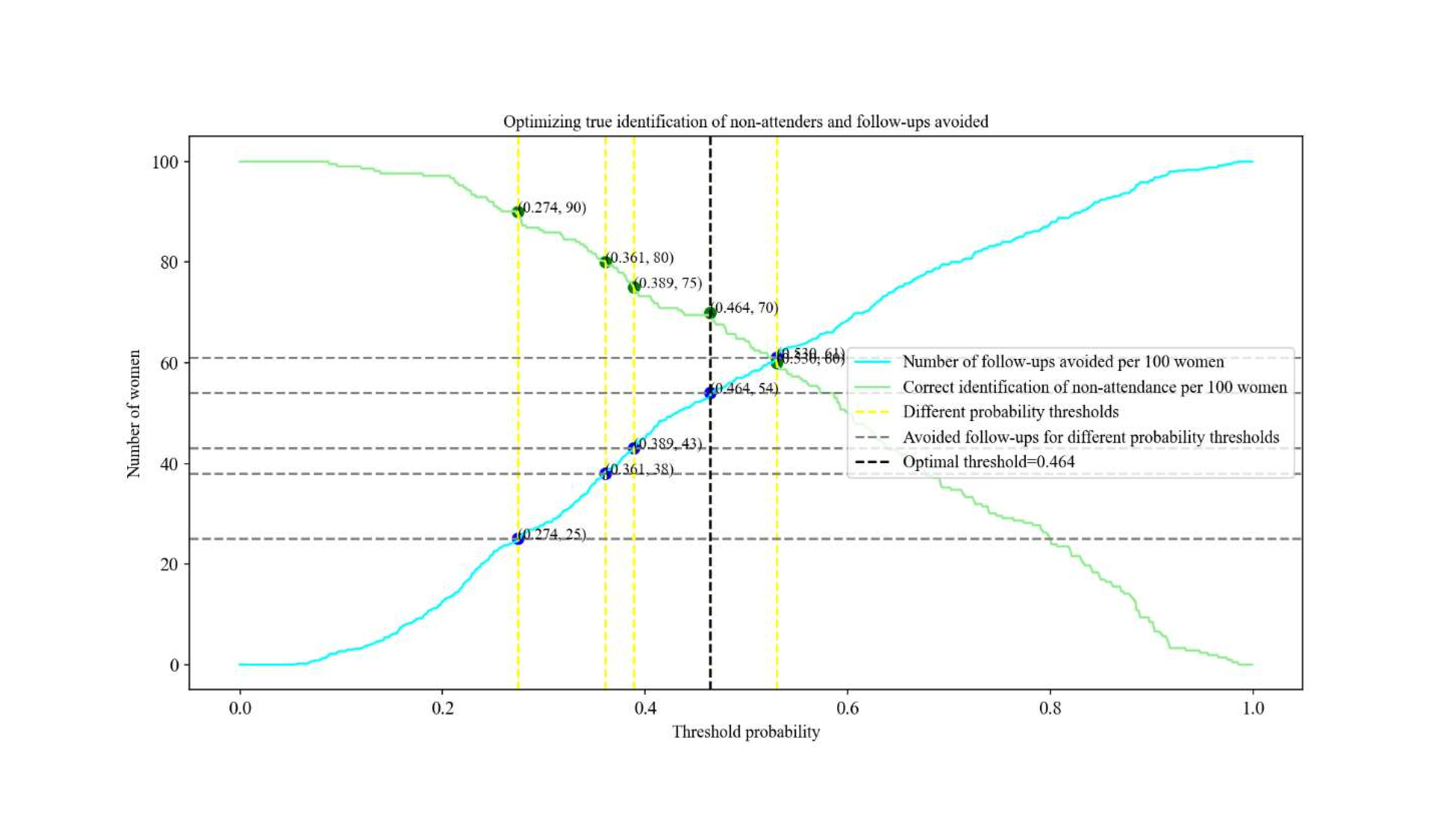

Supplement: S4 Fig — shows the comparison of correct prediction of non-attendance versus follow-ups avoided for different probability thresholds using our proposed model. Based on our proposed final model, the composite risk score, P(non-attendance), is calculated as 1/1+exp(-b), where b = -3.1599 + (0.1926*antenatal fasting glucose) + (-0.1415*antenatal postprandial glucose) + (0.0195*antenatal HbA1c) + (-0.0410*gestational age at antenatal OGTT) + (-0.0797*maternal age) + (0.0027*booking BMI) + (0.5486*parity) + (-0.8447*married) + (-0.2392*other ethnicity) + (0.8312*current smoker) + (0.0116*diastolic BP) + (0.1062*gestational age at birth) + (0.7418*women delivered SGA infants) + (0.4761*male sex of the baby) + (0.5630*instrumental delivery) + (-0.2115*initiated breastfeeding). SGA baby, smoker, marital status, male sex of baby, and other ethnicity are binary variables, taking value of either 0 or 1 depending on their absence or presence respectively. (TIF) [file pone.0264648.s004.tif]

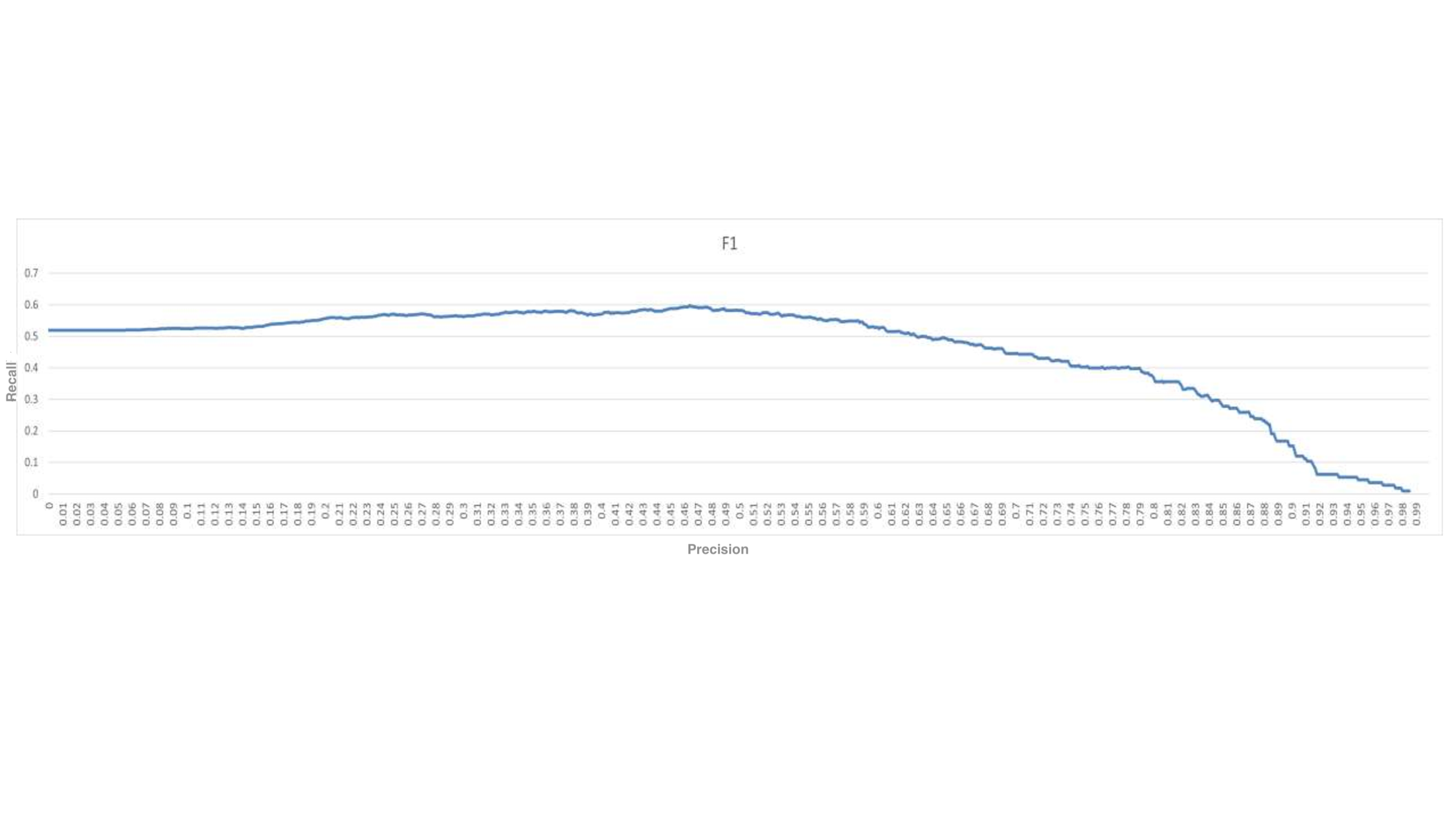

Supplement: S5 Fig — F1 graph showing the model sensitivity and specificity at variable thresholds. (TIF) [file pone.0264648.s005.tif]
