# Supplementary material for: Machine learning prediction of non-attendance to postpartum glucose screening and subsequent risk of type 2 diabetes following gestational diabetes
Source: PLoS One. 2022 Mar 7;17(3):e0264648. doi: 10.1371/journal.pone.0264648 (PMC8901061; doi:10.1371/journal.pone.0264648)
Supplement: S1 Table — (DOCX) [file pone.0264648.s006.docx]

**S1 Table: Cox proportional hazard ratio for dysglycaemia within 2 years of index pregnancy**

| **Predictors** | **β** | **SE** | **HR** | **95% CI for Exp(B)** | | **p-value** |
| --- | --- | --- | --- | --- | --- | --- |
|  |  |  |  | **Lower** | **Upper** |  |
| Antenatal HbA1c | 0.150 | 0.024 | 1.162 | 1.108 | 1.219 | <0.001^*^ |
| South Asian ethnicity | 0.881 | 0.393 | 2.414 | 1.118 | 5.212 | 0.025^*^ |
| Booking BMI | 0.078 | 0.026 | 1.081 | 1.028 | 1.137 | 0.003^*^ |
| Never smoked before or during pregnancy | -0.577 | 0.296 | 0.562 | 0.315 | 1.002 | 0.051 |

Cox proportional hazard model adjusted for multiple covariates including, ethnicity, antenatal smoking status, antenatal fasting and 2-hrs glucose, HbA1c, booking BMI, age, parity, systolic and diastolic blood pressure, gestational age at delivery and breastfeeding initiation stratified for ppGT attendance. ^*^p-value <0.05 was considered significant and calculated using Cox- proportional hazard model.
