# Supplementary material for: Machine learning prediction of non-attendance to postpartum glucose screening and subsequent risk of type 2 diabetes following gestational diabetes
Source: PLoS One. 2022 Mar 7;17(3):e0264648. doi: 10.1371/journal.pone.0264648 (PMC8901061; doi:10.1371/journal.pone.0264648)
Supplement: S1 Appendix — (PDF) [file pone.0264648.s007.pdf]

George Eliot Hospital
NHS Trust

George Eliot Hospital
College Street
Nuneaton CV10 7DJ
Telephone: (02476) 351351
Facsimile: (02476) 865175

Dear Madam

<u>Gestational Diabetes Post-Natal Blood Test</u>

You may remember during your recent pregnancy, you developed gestational diabetes. We recommend that you have a blood test following delivery of your baby to ensure that your blood glucose levels have returned to normal. Based on your results during pregnancy we advise that you have the following test:

- ☐ Glucose Tolerance Test- 6-12 weeks post delivery
- ☐ HbA1C- 3 months post delivery

Please find enclosed the relevant blood form.

Please ensure you make an appointment with your GP or practice nurse to obtain your results and further advice.

In addition, the following advice for the future is recommended:

1. If you plan to conceive or become pregnant, then be aware that you can develop diabetes again during pregnancy. If this is the case please make arrangements to visit your midwife or doctor immediately as you will require further tests.

2. You also have an increased risk of developing diabetes in the future and we advise you to maintain a healthy diet and lifestyle. Please see enclosed leaflet.

3. Due to this increased risk of developing diabetes, we also recommend you have a yearly HbA1C blood test.

If you develop any symptoms of diabetes you should be tested sooner and see your doctor immediately. These symptoms can include increased tiredness, thirst, and frequency in passing urine, blurred vision and thrush type infections. If you experience any of these or are in any doubt please see your doctor or nurse.

Yours sincerely

Prof Saravanan	J Plester	J Wilson / S Selvamoni	T Ritchie
Consultant	Diabetes Midwife	Diabetes Specialist Nurses	Diabetes Associate Practitioner
