# Supplementary material for: Machine learning prediction of non-attendance to postpartum glucose screening and subsequent risk of type 2 diabetes following gestational diabetes
Source: PLoS One. 2022 Mar 7;17(3):e0264648. doi: 10.1371/journal.pone.0264648 (PMC8901061; doi:10.1371/journal.pone.0264648)
Supplement: Attachment — Submitted filename: Reviewers comment.docx [file pone.0264648.s010.docx]

**Reviewer’s comment**

In the study titled ‘Machine learning prediction of nonattendance to postpartum glucose screening and subsequent risk of type 2 diabetes following gestational diabetes,’ the authors Periyathambi et al. have attempted to find the characteristics/habits of the women who did not attend postpartum glucose testing (ppGT) despite the reminder from the hospital staff. This is a retrospective cohort study of all 607 GDM women who were ppGT due between January 2016 and December 2019 at the George Eliot Hospital NHS Trust, UK. This study indicates that the women who did not attend ppGT were younger, multiparous, obese, and continued smoking during pregnancy. Most of these characteristics of individuals are comparable with their previous study (Venkararaman et al., 2015). The authors have used machine learning (Python version 3.7) for predicting the type of women who were more likely to have nonattendance for ppGT. This study has many limitations such as small sample size, uniformity in reminding the individuals, etc. However, this preliminary study can encourage researchers to do a study on a larger sample size in the future. Following are a few suggestions that might improve the article.

1. The authors may explain how different machine learning results are compared to other analyses such as manual analysis.
2. The in-person consultation with hospital staff at the time of discharging from the hospital after delivery can play an important role in whether the woman will attend ppGT or not. Also, the amount of importance given for the consultation may vary among hospital staff.
3. The authors may consider including a future direction such as ‘A study with larger sample size would give better clarity on machine learning for nonattendance for ppGT.
