# Supplementary material for: Machine learning prediction of non-attendance to postpartum glucose screening and subsequent risk of type 2 diabetes following gestational diabetes
Source: PLoS One. 2022 Mar 7;17(3):e0264648. doi: 10.1371/journal.pone.0264648 (PMC8901061; doi:10.1371/journal.pone.0264648)
Supplement: Attachment — Submitted filename: Review_Periyathambi et al_PONE-D-21-35154.docx [file pone.0264648.s011.docx]

The manuscript by Periyathambi et al, titled " *Machine learning prediction of non-attendance to postpartum glucose screening and subsequent risk of type 2 diabetes following gestational diabetes*" studies the social and physiological factors associated with non-attendance of immediate postpartum glucose test using machine learning algorithm in women with gestational diabetes mellitus.

The study also presents correlation between non-attendance and higher risk of conversion to type 2 diabetes mellitus.

The study is well designed and executed and the import of the report is evident in its findings. However, I would like the authors to discuss the effectiveness of their machine learning model and its predictive power in a different socio-economic setting – for example in under-developed or developing societies. This would certainly enhance the quality and relevance of the manuscript.

Overall, the authors did an excellent job in organizing their workflow, and the manuscript is easily understandable even for non-expert readers. I applaud the authors for their commendable effort.
