# Supplementary material for: Machine learning prediction of non-attendance to postpartum glucose screening and subsequent risk of type 2 diabetes following gestational diabetes
Source: PLoS One. 2022 Mar 7;17(3):e0264648. doi: 10.1371/journal.pone.0264648 (PMC8901061; doi:10.1371/journal.pone.0264648)
Supplement: Attachment — Submitted filename: Response to Reviewers_Final.docx [file pone.0264648.s012.docx]

Dr Emily Chenette

Editor-in-chief

PLOS ONE

Submission Date: 03/11/2021

Dear Editor,

Thank you for inviting us to submit a revised draft of our manuscript, PONE-D-21-35154, entitled “**Machine learning prediction of non-attendance to postpartum glucose screening and subsequent risk of type 2 diabetes following gestational diabetes**”. We thank the reviewers for their insightful comments and appreciate the time and effort you and each of the reviewers have committed to providing suggestions to improve our manuscript. In the revised manuscript we have adhered to PLOS ONE journal requirement suggestions that reflects detailed revision on style requirements, funding disclosure and anonymous data sharing. We have also incorporated the feedback and critiques that the reviewers have suggested.

**EDITOR SUGGESTIONS:**

1. Please ensure that your manuscript meets PLOS ONE's style requirements, including those for file naming.

**Response:**

Thank you for your suggestion to follow the PLOS ONE style throughout the manuscript. We have incorporated the changes in the ‘Revised Manuscript with Track Changes’.

2. Please provide an amended statement that declares *all* the funding or sources of support (whether external or internal to your organization) received during this study, as detailed online in our guide for authors at <http://journals.plos.org/plosone/s/submit-now>.

**Response:**

As recommended by the editors, we have incorporated our funding disclosure in the revised statement as follows,

- PS and YW are part funded by Medical Research Council, UK Grant number:  MR/R020981/1

Funder: MRC- UK

URL: <https://mrc.ukri.org/funding/>

- NP is funded by Chancellor's international Scholarship for doctoral research.
  Funder: University of Warwick
  URL: <https://warwick.ac.uk/services/dc/schols_fund/scholarships_and_funding/chancellors_int/>
- RS is supported by his institution, funded by the Department of Atomic Energy, Government of India.

We declare that the funders had no role in study design, data collection and analysis, decision to publish, or preparation of the manuscript and, there was no additional external funding received for this study.

3. Please clarify whether this conference proceeding was peer-reviewed and formally published. If this work was previously peer-reviewed and published, in the cover letter please provide the reason that this work does not constitute dual publication and should be included in the current manuscript.

**Response:**

We would like to clarify that our abstract published in the ‘56^th^ EASD Annual Meeting of the European Association for the Study of Diabetes’ constitutes statistical analysis of complete cases of n=551 study participants. We declare that we have not published any premature works of machine learning model and/or the additional analysis, i.e., decision curve analysis and two-year follow-up study findings in the conference. Therefore, we believe that our conference presentation cannot be considered as prior publication. Also, we would like to highlight an accessible URL to the published abstract for your reference.

Title: Improving the uptake of postpartum glucose screening following a gestational diabetes pregnancy

URL: <https://link.springer.com/article/10.1007/s00125-020-05221-5> ; Abstract Number: 652

4. In your revised cover letter, please address the following prompts:

a) If there are ethical or legal restrictions on sharing a de-identified data set, please explain them in detail

(e.g., an ethics committee). Please also provide contact information for a data access committee, ethics committee, or other institutional body to which data requests may be sent.

b) If there are no restrictions, please upload the minimal anonymized data set necessary to replicate your study findings as either Supporting Information files or to a stable, public repository and provide us with the relevant URLs, DOIs, or accession numbers.

**Response:**

We agree with you regarding the data sharing policy. Hence, we have reflected this suggestion by uploading the anonymized data set as supporting information csv file as S3 Appendix.csv. Full dataset is available on request following completion of suitable confidentiality agreement. We have highlighted the same in the ‘Revised Manuscript with Track Changes’ in page 21, lines 509:511.

**REVIEWER 1 COMMENTS:**

5. It would be very helpful to further extend the discussion to include what other factors the current model is not taking in consideration, and which may be very interesting to improve the accuracy of the prediction (My first thought goes to the proximity to a testing center, the flexibility in having an appointment, the nature of jobs that these individuals have...etc. How this can be addressed in future studies, etc.

**Response:**

We thank the reviewers for this important point.

- We agree that factors such as access to nearest healthcare centre and travel distance could impact women’s participation in screening (Cullinan *et al*, 2011). However, all the study participants in our cohort had easy access to the testing centre (the catchment area is small, less than 5 miles for those who live in the city and easy/free parking at the hospital). They all are given the option to choose their appointments too if the offered appointment date/time is not suitable. We acknowledge that our anonymous data collection process could not incorporate otherwise identifiable records, i.e personal address and postcode.
- We have supplemented the S1 Appendix section of the invite that implies flexibility to have an appointment at the client’s earliest availability. In addition, all the participants had telephone reminders regarding their appointment booking by one of the specialist nurse practitioners where it provides an opportunity for the women to amend their screening date and/or time.
- Although we found higher proportion of employed women (64.8%) attending the postpartum glucose testing compared to unemployed women (50%), it was not statistically significant. We have also incorporated our clarification in discussion in page 15, lines 335:341. We agree further research on the barriers and facilitators of postpartum glucose testing is needed to provide additional insights.

6. The font is not homogenous in the discussion section and need to be adjusted.

**Response:**

We have addressed this comment by modifying the font size to 11pts in page, 11:12 and a spacing in line 318 on page 14.

**REVIEWER 2 COMMENTS:**

7. Authors should highlight significant values in tables with asterisk marks or underline etc., for younger, unmarried, multiparous, BMI and smoking during pregnancy.

**Response:**

Thank you for this suggestion. We have incorporated this comment by including a separate column for p-values and asterisk marks in the ‘Characteristics’ column to show significance in Table.1 in ‘Revised Manuscript with Track Changes’.

1. Authors should describe the details of model analysis in the methods section. Readers benefited by including the S1 file which mentioned "Stepwise 1 description of the Machine learning" into methods section.

**Response:**

We agree with your comments and have incorporated this suggestion in the Method section in page 6:7, lines 150:168.

2. a) Results sections of the study were not well explained, what is the concept diagram used in this study, how does it generate helpful outcomes of the Figure S2 for next steps.

b) The area under ROC was 0.72. How does this value help to decide the factors associated with non-attendance of glucose tests?

c) The outcome results of decision curve analysis is not explained in detail.

d) How does decision curve analysis can be used to find factors or glucose levels in women who did not attend gestational type 2 diabetes (T2D) screening in the postpartum period.

**Response:**

a) We would like to clarify how the concept diagram used in this study as follows: The processed data is divided into 5 folds. The light grey region shows the training data, and the dark grey shows the testing data. Within each iteration *i* of the outer 5-fold CV, the training folds further undergo internal 10-fold CV in Step 1 for lasso hyperparameter optimization. This is known as nested cross validation. In step 2, feature selection is performed on the training folds in iteration *i* using lasso with optimized regularization hyperparameter. Logistic regression model with selected features is fit on the training folds in iteration *i* in Step 3. The fit model is used for prediction on the exclusively held out test data in iteration *i*. The test predictions from all 5 iterations are aggregated to plot and calculate the area under the ROC curve for evaluating the performance of our method. We have updated the figure caption to explain this.

b) A non-informative classifier which picks the label at random, will have a 0.5 area under the ROC (AUROC), while a perfect one will have an AUROC of 1.0. A value of 0.72 on an unseen (test) dataset denotes that the classifier is informative, although not perfect. Different classifiers will result in different areas, the 0.72 was the best possible area among the methods we tried. The factors that give the highest area under the curve on an unseen dataset are listed as the most prominent factors associated with non-attendance of glucose tests.

c) We have redrafted the result section by incorporating decision curve analysis (DCA) outcome with the optimal threshold (0.464) to target non-attenders based on our prediction model in page 12, lines 248: 250 to be more in line with your comments. The DCA has higher net benefit than the conventional strategy to focus all the women or targeting none which would lead to improved glucose screening rate in any clinical settings. We have also updated the Fig.3 legend to show the DCA comparisons between the prediction model vs ‘target all’ or ‘target none’ lines in page 13, lines 260:261.

d) The DCA evaluated our prediction model which encompasses all the variables that were used to construct the final model (antenatal OGTT values, i.e, fasting glucose and 2-hrs glucose and gestational age at the time of screening, antenatal HbA1c, age, booking BMI, parity, marital status, ethnicity, smoking status, booking BP, gestational age at birth, infant birth size and sex, type of delivery and breastfeeding initiation assessed before the hospital discharge). Therefore, our DCA has overcome the limitation of discriminating women who would not have been screened for gestational diabetes and otherwise targeted via other predictive factors. We would like to further address your comments by highlighting the S2 Appendix, an Excel based calculator which shows the combination of all the predictive factors to identify women who are less likely to attend the ppGT. In simple terms, DCA was not implemented to find factors associated with non-attendance; DCA helps to understand that the classifier built with the chosen factors (corresponding to highest AUROC) is more beneficial than follow-up of all GDM women assuming none will attend.

3. Authors should provide any previous examples or references for the machine learning (ML) used in this study. How does this ML help in the example disease predictive models?

**Response:**

Thank you for your suggestion. We have included a reference for machine learning approach to predict a disease (Type 2 Diabetes after GDM pregnancy) in page, 6, line number 134. However, while the basic methods such as logistic regression and lasso are standard, the detailed framework as presented here is our own, and approaches like nested cross validation to find optimal hyper-parameters have not been used before in such studies, to the best of our knowledge.

4. Authors mention in line 148 “After getting assurance of acceptable performance of this method” how does the assurance of method was confirmed by authors in this study, please explain features that help to get confidence of the model.

**Response:**

The term “acceptable performance” refers to the AUROC of 0.72 over the unseen data. A value consistently higher than 0.7 i.e., better than the 0.5 a random classifier would achieve assures us of its performance. Along with AUROC, high sensitivity as well as specificity together help to get confidence from the model.

5. Authors should report how hospitals can be improved the personalised education for postpartum glucose screening using this study, whether this can be applied to hospitals in many countries or specific to a country. How it can be improved could be described in discussion or if any country has better screening for gestational type 2 diabetes (T2D) in the postpartum period, additional details of these elements could be helpful to improve the study or personalised education.

**Response:**

We have elaborated the usefulness of our ML predictive model in better understanding of personalised education that could improve the ppGT uptake rate in page 14, lines 298:301. We hope these revisions provide a more balanced discussion on the implementation of individualised education in addition to the reminder or recall systems described in few previous studies, Benhalima et al (2017) and Carmody et al (2015).

6. Add a short form for the area under the ROC as AUROC for figure2 legend or results section describe figure2.

**Response:**

We have addressed your suggestion in page 6 in line 145 and in page 11 in line 225 to rewrite the short form of area under the ROC.

7. Label x and y axis in fig S5.

**Response:**

We have addressed this comment by including ‘x’ and ‘y’ axis label as ‘Precision’ and ‘Recall’ for the S5 Fig.

**REVIEWER 3 COMMENTS:**

8. The authors may explain how different machine learning results are compared to other analyses such as manual analysis.

**Response:**

We are unsure of what the reviewer means by manual analysis. We assume the reviewer means standard statistical analysis. ML methods used here are of the predictive kind, where the eventual goal is to predict the class of a new case (not seen thus far by the model). Statistical analyses will identify differences across individual predictors, between the classes (the p-value listed in Table.1), but do not give a score or a prediction based on them. We note that the final formula learned by the method, that does the best among all methods we tried, can in fact be applied using a simple calculator (as provided in the S2 Appendix) by the healthcare professional at his/her own clinic.

9. The in-person consultation with hospital staff at the time of discharging from the hospital after delivery can play an important role in whether the woman will attend ppGT or not. Also, the amount of importance given for the consultation may vary among hospital staff.

**Response:**

We agree with you and have incorporated this suggestion in page 14, lines 309:311.

10. The authors may consider including a future direction such as ‘A study with larger sample size would give better clarity on machine learning for nonattendance for ppGT.

**Response:**

Thank you for your suggestion and we have revised the text in page 15, lines 353:355 to address the usefulness of ML prediction model.

**REVIEWER 4 COMMENTS:**

11. The study is well designed and executed, and the import of the report is evident in its findings. However, I would like the authors to discuss the effectiveness of their machine learning model and its predictive power in a different socio-economic setting – for example in under-developed or developing societies. This would certainly enhance the quality and relevance of the manuscript.

**Response:**

We have incorporated your comments in page 14:15, lines 325:327 to reflect the easily identifiable factors for the evaluation of non-attenders for postpartum screening. Our Excel based calculator- S2 Appendix could be used in any low resource setting to target women who are less likely to turn up for the postpartum screening.
